# Digital PCR applications for the diagnosis and management of infection in critical care medicine

**DOI:** 10.1186/s13054-022-03948-8

**Published:** 2022-03-21

**Authors:** Irene Merino, Amanda de la Fuente, Marta Domínguez-Gil, José María Eiros, Ana P. Tedim, Jesús F. Bermejo-Martín

**Affiliations:** 1 Group for Biomedical Research in Sepsis (BioSepsis), Instituto de Investigación Biomédica de Salamanca, (IBSAL), Paseo de San Vicente, 58-182, 37007 Salamanca, Spain; 2 Hospital Universitario Río Hortega, Calle Dulzaina, 2, 47012 Valladolid, Spain; 3 Microbiology Department, Hospital Universitario Río Hortega, Calle Dulzaina, 2, 47012 Valladolid, Spain; 4 Centro de Investigación Biomédica en Red en Enfermedades Respiratorias (CIBERES), Instituto de Salud Carlos III, Av. de Monforte de Lemos, 3-5, 28029 Madrid, Spain

**Keywords:** Critically ill patients, Digital PCR, Infection diagnosis, Prognosis and treatment guidance, Host response

## Abstract

Infection (either community acquired or nosocomial) is a major cause of morbidity and mortality in critical care medicine. Sepsis is present in up to 30% of all ICU patients. A large fraction of sepsis cases is driven by severe community acquired pneumonia (sCAP), which incidence has dramatically increased during COVID-19 pandemics. A frequent complication of ICU patients is ventilator associated pneumonia (VAP), which affects 10–25% of all ventilated patients, and bloodstream infections (BSIs), affecting about 10% of patients. Management of these severe infections poses several challenges, including early diagnosis, severity stratification, prognosis assessment or treatment guidance. Digital PCR (dPCR) is a next-generation PCR method that offers a number of technical advantages to face these challenges: it is less affected than real time PCR by the presence of PCR inhibitors leading to higher sensitivity. In addition, dPCR offers high reproducibility, and provides absolute quantification without the need for a standard curve. In this article we reviewed the existing evidence on the applications of dPCR to the management of infection in critical care medicine. We included thirty-two articles involving critically ill patients. Twenty-three articles focused on the amplification of microbial genes: (1) four articles approached bacterial identification in blood or plasma; (2) one article used dPCR for fungal identification in blood; (3) another article focused on bacterial and fungal identification in other clinical samples; (4) three articles used dPCR for viral identification; (5) twelve articles quantified microbial burden by dPCR to assess severity, prognosis and treatment guidance; (6) two articles used dPCR to determine microbial ecology in ICU patients. The remaining nine articles used dPCR to profile host responses to infection, two of them for severity stratification in sepsis, four focused to improve diagnosis of this disease, one for detecting sCAP, one for detecting VAP, and finally one aimed to predict progression of COVID-19. This review evidences the potential of dPCR as a useful tool that could contribute to improve the detection and clinical management of infection in critical care medicine.

**Supplementary Information:**

The online version contains supplementary material available at 10.1186/s13054-022-03948-8.

## Introduction

### Infection in critical care medicine

Infectious pathology represents a leading cause of admission to the intensive care units (ICU). Sepsis (defined by the presence of a dysregulated host response to infection inducing organ dysfunction) is present in up to 30% of all ICU patients, as recently reported by Sakr et al. in a large study with 10,000 patients from 730 ICUs [1]. One of the leading causes of sepsis is severe community acquired pneumonia (sCAP) of bacterial or viral origin [2]. Current Coronavirus disease 2019 (COVID-19) pandemics has largely boosted the cases of sCAP all over the world.

In turn, infection is one of the most frequent complications in patients who are critically ill. Compromise of body’s physical barriers by invasive devices, surgical aggression or traumatic injury, disruption of the mucosa, pressure sores, ventilator-induced lung injury, immune suppression, poor nutritional state, the use of broad-spectrum antibiotics which alter the commensal microbiota, combined with the increased exposition to opportunistic (often multi-drug resistant, MDR) pathogens [3], all represent predisposing factors favouring ICU acquired infections [4]. In fact, approximately 19.2% of ICU patients develop infections compared to approximately 5.2% of infections developed by patients staying in all other hospital wards [3, 5]. Ventilator-associated pneumonia (VAP) affects 10–25% of all ventilated patients after at least 48 h on mechanical ventilation [6]. Other frequent nosocomial infections affecting critically ill patients are catheter-associated urinary tract infection, bloodstream infection (BSIs), skin and wound infections, sinusitis, and gastrointestinal infection (often with *Clostridium difficile*) [4]. Clinical management of these infectious diseases or complications of the critically ill patient faces several challenges, including early diagnosis with microorganism identification, severity stratification, prognosis assessment and treatment guidance. Digital polymerase chain reaction (dPCR) is a next-generation PCR method that represents an opportunity to address these challenges.

### dPCR: technical principles and applications

dPCR has emerged as a promising technology that might fill in the current gaps of other standard or emerging diagnostic technologies employed in microbiology (Table S1 – additional file 1). dPCR is based on the division of the PCR mastermix (all components including DNA or RNA targets) into thousands of partitions. PCR amplification of target genes occurs in each individual partition, acting as an individual microreactor [7]. These partitions can be created using a number of different mechanisms, such as emulsified microdroplets suspended in oil (droplet digital PCR, ddPCR), manufactured microwells, or microfluidic valving [8]. The distribution of target sequences in the partitions is detected by fluorescence at endpoint. Quantification of target genes is estimated based on Poisson’s distribution, by calculating the ratio of positive partitions (presence of fluorescence) over the total number of partitions [9]. This technology has several advantages: i) it is less affected by PCR inhibitors than other standard or real-time PCR (qPCR) methods, as target sequences are concentrated in the microreactors; ii) it also offers a high reproducibility of the results; iii) it provides an absolute quantification of the target sequence without the need for standard curves; and iv) it has an improved analytical sensitivity ideal for detecting microbial genes, for species identification or for genes conferring antimicrobial resistance or higher pathogenicity. dPCR also presents some limitations: i) it is unable to distinguish between viable and non-viable microorganisms (an inconvenient which affects all PCR-based methods); ii) it might have different sensitivity for different types of microorganisms; iii) it needs specialized training; and iv) it has a high cost, particularly to acquire the devices. This represents a major drawback for applications in low or middle income countries, for example during the COVID-19 pandemics [7, 9]. In spite its limitations, the previously mentioned dPCR properties make it an ideal tool for clinical applications in the field of microbiology and infectious diseases [7]. In this article we reviewed the existing evidence on the use of dPCR to improve the clinical management of infection in critical care medicine.

## Methods

**Fig. 1:**
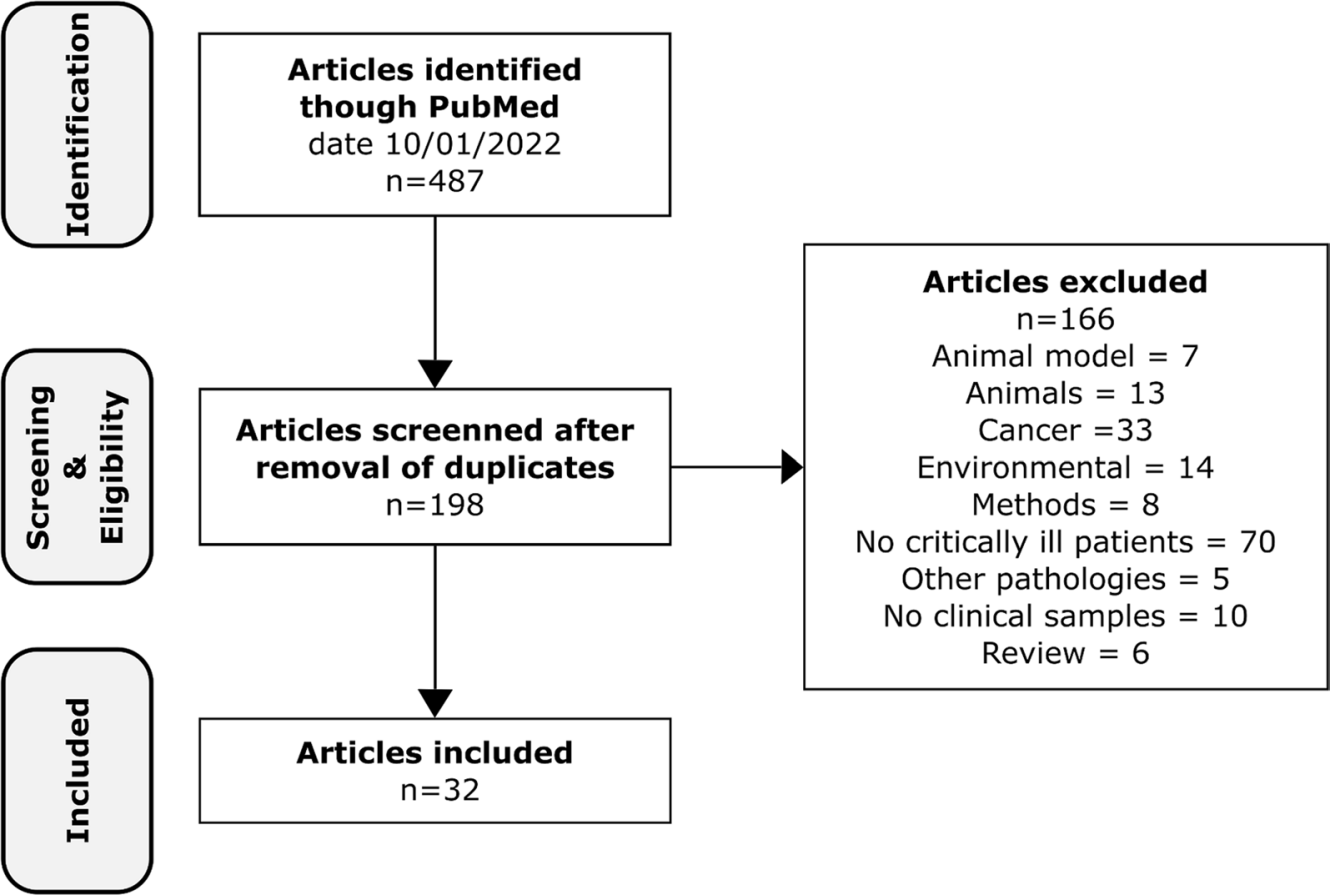
PRISMA diagram

We searched PudMed combining the following MESH terms: “digital PCR”, “digital droplet PCR”, “droplet digital PCR” and “droplet PCR” with “ICU”, “critical AND infection”, “critically ill AND infection”, “severe infection”, “critical care”, “pneumoniae”, “ventilator-associated pneumoniae”, “ventilator”, “ventilation”, “sepsis”, “septic shock”, “bloodstream infections”, “skin and soft tissue infections”, “necrotizing fasciitis”, “peritonitis”, “invasive pulmonary aspergillosis”. We found a total of 487 PubMed articles. Only articles in English were considered. Using PMID we excluded duplicated articles appearing in more than one search, obtaining a total of 198 articles. We screened these articles for relevance and excluded 166 for the reasons mentioned in Fig. 1. Finally, thirty-two articles were included in this review (Table 1). These articles were further divided in two groups, one focused on the pathogens—diagnosis of infection, prognosis and treatment guidance (*n* = 23)—and the other focused on the host response to infection (*n* = 9).

**Table 1:** Articles included in the review depicting the applications of dPCR for infection diagnosis and management in critical care medicine

Study	Year	Usage	Entity	Objective	References
Hu et al.	2021	Bacterial identification in blood or plasma	Sepsis	Identification of DNA from bacterial pathogens and antimicrobial resistance genes in blood from patients BSI	[10]
Yamamoto et al.	2018	Bacterial identification in blood or plasma	Sepsis	Identification of *Mycobacterium tuberculosis* through the detection of circulating cell-free DNA	[14]
Shin et al.	2021	Bacterial identification in blood or plasma	BSI	Diagnosis of Gram-Negative pathogens and antimicrobial resistance genes in plasma from patients with BSIs	[15]
Zheng et al.	2021	Bacterial identification in blood or plasma	Sepsis	Identification of DNA from *Acinetobacter baumannii* and *Klebsiella pneumoniae* in blood from patients BSI	[16]
Chen et al.	2021	Fungal identification in blood	BSI	Diagnosis of *Candida* spp in blood from patients with BSI	[18]
Zhou et al.	2021	Bacterial and fungal identification in other clinical samples	Pleural or peritoneal infections	Identification of pathogens from pleural or peritoneal infections	[19]
Simms et al.	2021	Viral identification	COVID-19	Confirmation of detection of SARS-CoV-2 in renal allograft and lung tissue (initially detected by immunohistochemistry)	[20]
Alteri et al.	2020	Viral identification	COVID-19	Quantification of SARS-CoV-2 viral load in plasma of patients with negative qPCR results	[21]
Jiang et al.	2020	Viral identification	COVID-19	Quantification of SARS-CoV-2 in plasma and hospital environment	[22]
Ziegler et al.	2019	Quantification of microbial burden to assess severity, prognosis and treatment guidance	Sepsis	Quantification of DNA load overtime in patients with *Staphylococcus aureus* BSIs	[29]
Ziegler et al.	2019	Quantification of microbial burden to assess severity, prognosis and treatment guidance	Sepsis	Quantification of DNA from bacterial pathogens load (16S rDNA) overtime in patients BSI to access patients’ progression	[30]
Bialasiewicz et al.	2019	Quantification of microbial burden to assess severity, prognosis and treatment guidance	Sepsis	Quantification of DNA in blood from *Capnocytophaga canimorsus* to access patients progression	[31]
Dickson et al.	2020	Quantification of microbial burden to assess severity, prognosis and treatment guidance	VAP	Quantification of bacteria DNA burden in the lung and association with disease progression and outcomes	[32]
Goh et al.	2020	Quantification of microbial burden to assess severity, prognosis and treatment guidance	Sepsis sCAP	Quantification of EBV (to detect reactivation) in patients with sepsis to monitor disease progression	[33]
Veyer et al.	2021	Quantification of microbial burden to assess severity, prognosis and treatment guidance	COVID-19	Quantification of SARS-CoV-2 viral load in plasma and correlation with disease severity	[34]
Chen et al.	2021	Quantification of microbial burden to assess severity, prognosis and treatment guidance	COVID-19	Quantification of SARS-CoV-2 viral load in plasma and correlation with disease severity	[35]
Bermejo-Martin et al.	2020	Quantification of microbial burden to assess severity, prognosis and treatment guidance	COVID-19	Quantification of SARS-CoV-2 viral load in plasma and correlation with disease severity	[36]
Ram-Mohan et al.	2021	Quantification of microbial burden to assess severity, prognosis and treatment guidance	COVID-19	Quantification of SARS-CoV-2 viral load in plasma and correlation with disease severity	[37]
Tedim et al.	2021	Quantification of microbial burden to assess severity, prognosis and treatment guidance	COVID-19	Quantification of SARS-CoV-2 viral load in plasma, comparison with qPCR	[38]
Martin-Vicente et al.	2022	Quantification of microbial burden to assess severity, prognosis and treatment guidance	COVID-19	Quantification of SARS-CoV-2 viral load in plasma	[39]
Bruneau et al.	2021	Quantification of microbial burden to assess severity, prognosis and treatment guidance Host Response	COVID-19	Quantification of SARS-CoV-2 viral load and host biomarkers in plasma to predict disease severity	[40]
Chanderraj et al.	2022	Microbial ecology studies	Sepsis	Quantification of bacterial density in rectal swabs and risk of extraintestinal infection	[41]
Brooks et al.	2018	Microbial ecology studies	Microbiological burden and microbiome	Quantification of total microbiological burden in hospital neonates ICU and correction with microbiome establishment	[42]
Tamayo et al.	2014	Host Response	Sepsis	Quantification of the expression of the constant region of the mu heavy chain of IgM in blood to differentiate sepsis from SIRS	[46]
Almansa et al.	2019	Host Response	Sepsis	Gene expression ratio between MMP8 or LCN2 with HLA-DRA to differentiate surgical patients with sepsis from those with no sepsis	[47]
Almansa et al.	2018	Host Response	Sepsis	Ratio between HLA-DRA expression and procalcitonin to differentiate surgical patients with sepsis from those with no sepsis	[48]
Link et al.	2020	Host Response	Sepsis	Quantification of miRNA in blood for the early diagnosis of sepsis	[49]
Martin-Fernandez et al.	2020	Host Response	Sepsis	Quantification of emergency granulopoiesis gene expression to stratify severity in patients with infection, sepsis and septic shock	[50]
Menéndez et al.	2019	Host Response	sCAP	Gene expression levels of the immunological synapse genes to identify patients with sCAP	[52]
Almansa et al.	2018	Host Response	VAP	Gene expression levels of the immunological synapse genes to identify VAP	[53]
Busani et al.	2020	Host Response	Sepsis	Quantification of mtDNA in patients with Septic shock cause by MDR pathogens predicts disease severity	[54]
Sabbatinelli et al.	2021	Host Response	COVID-19	Quantification of miRNA associated with inflammation and aging (Inflammaging) to predict COVID-19 disease progression	[56]

DNA, deoxyribonucleic acid; BSIs, bloodstream infections; spp, Species; COVID-19, Coronavirus disease 2019; SARS-CoV-2, Severe acute respiratory syndrome coronavirus 2; qPCR, real-time polymerase chain reaction; VAP, ventilator-associated pneumoniae; sCAP, severe community acquired pneumoniae; EBV, Epstein Barr Virus; rDNA, ribosomal deoxyribonucleic acid; ICU, intensive care unit; IgM, immunoglobulin M; SIRS, Systemic inflammatory response syndrome; MMP8, matrix metalloproteinase-8; LCN2, Lipocalin 2; HLA-DRA, major histocompatibility complex class II; miRNA, micro ribonucleic acid; mtDNA, mitochondrial deoxyribonucleic acid; MDR: multidrug-resistant

## Evidence on the use of dPCR for the diagnosis and management of infection in critical care medicine (Fig. 2)

**Fig. 2:**
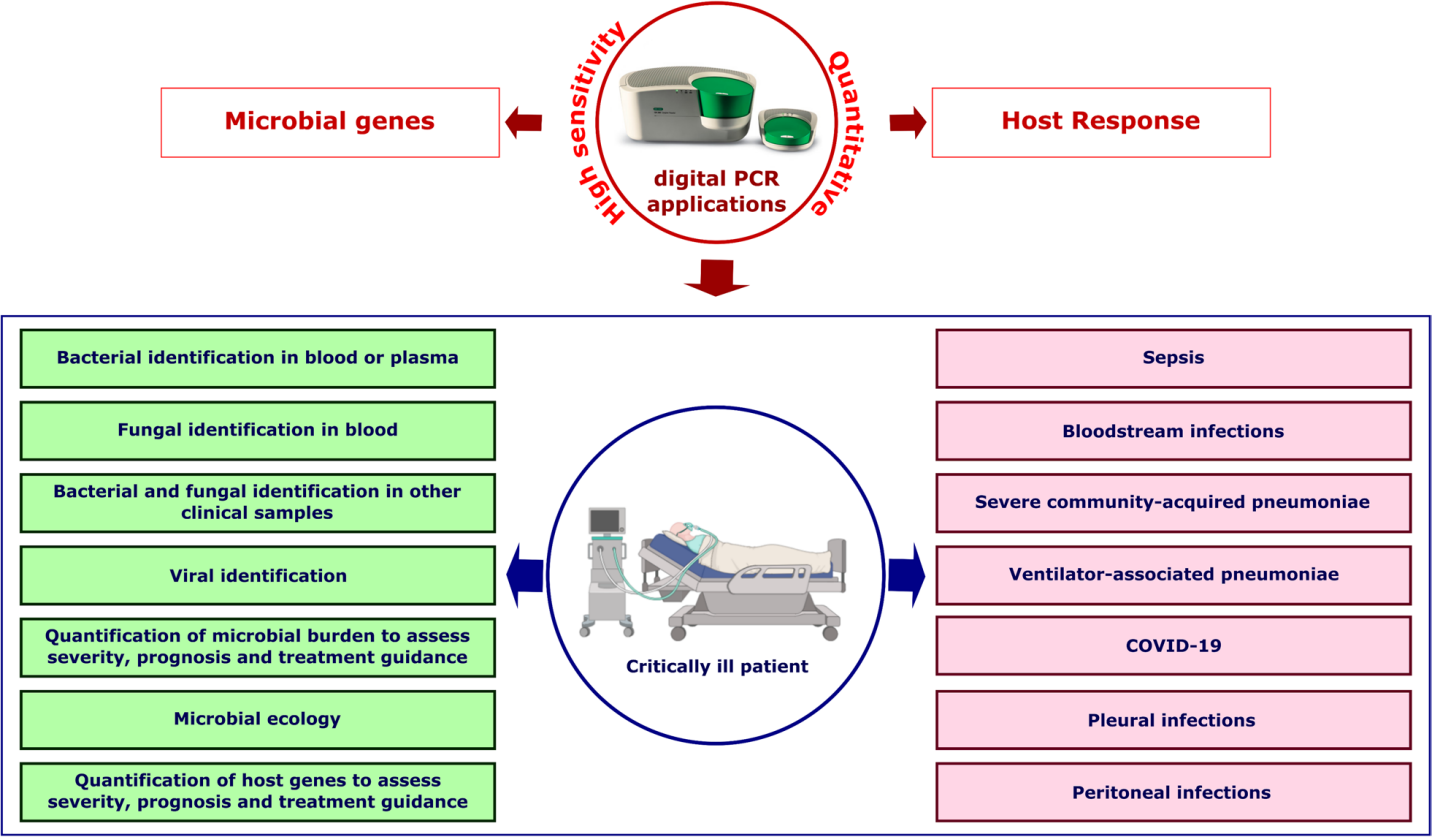
Summary of the existing evidence on the applications of dPCR in the field of infection in Critical Care Medicine

### Applications of dPCR targeting microbial genes

The gold standard for the detection of bacterial and fungal pathogens still relies on culture based methods that present a long turnaround time, and often yield low positivity rates [10]. For viral pathogens the reference method is frequently qPCR [11–13]. As previously stated, dPCR presents several advantages making it an ideal technique for the detection and quantification of microbial genes (Additional file 1: Table S1).

#### Bacterial identification in blood or plasma

**Fig. 3:**
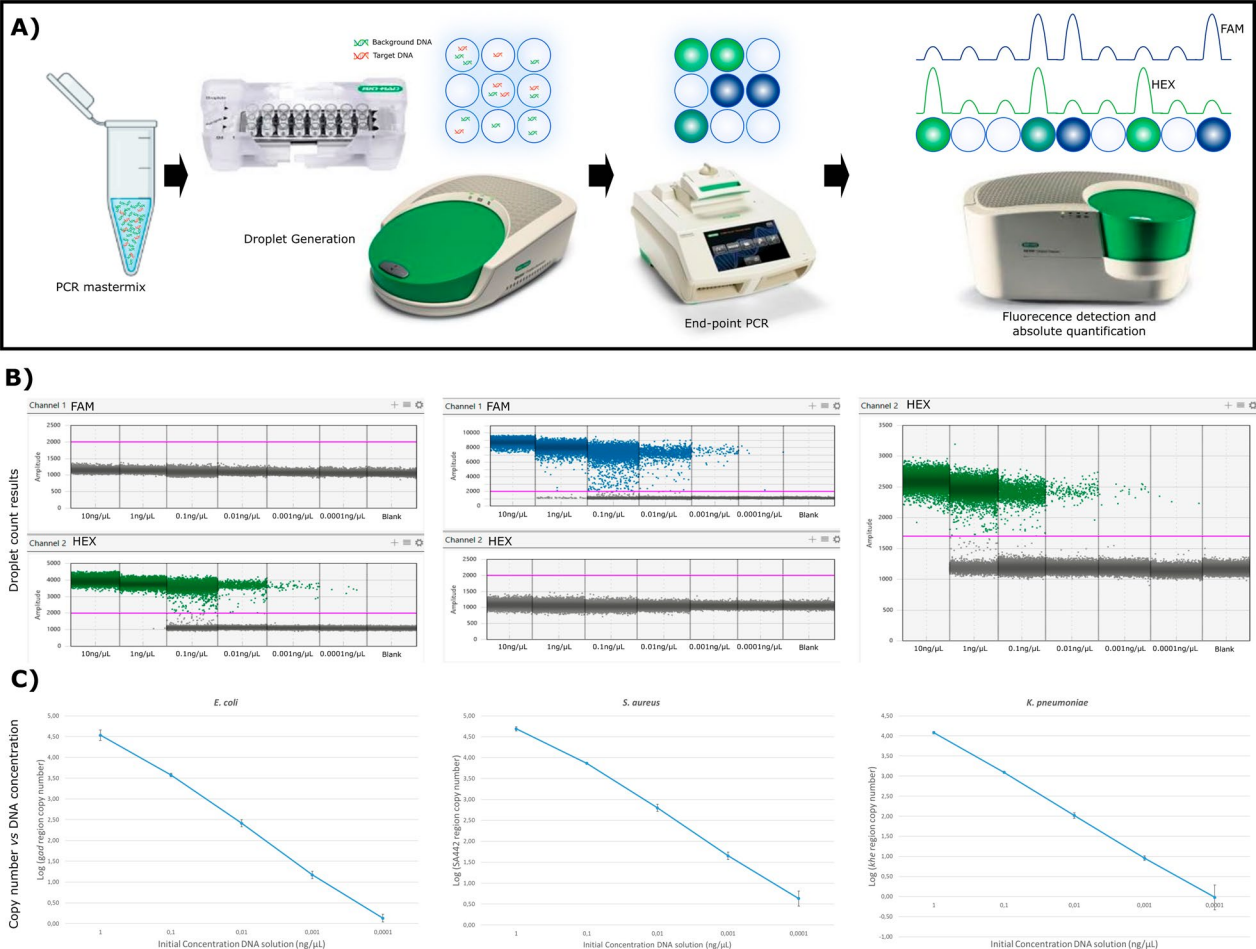
dPCR detection assay for *E. coli*, *K. pneumoniae* and *S. aureus*. **A** ddPCR workflow; **B** ddPCR results displayed as droplets of different fluorescence amplitude; **C** copy number of *E. coli*, *S. aureus* and *K. pneumonia*e in different DNA of different initial DNA concentration

dPCR has been used in critical care medicine for the detection of different bacterial pathogens in septic patients or patients with a suspected BSI [10, 14–16]. Yamamoto et al. successfully diagnosed a septic patient with a *Mycobacterium tuberculosis* (MTB) disseminated infection by detecting MTB complex-specific sequences in total cell-free DNA (cfDNA) in plasma. dPCR was employed after sputum, urine, and blood samples all tested negative by COBAS TaqMan MTB and MAI tests (Roche Diagnostics) and TSPOT.TB test (Oxford Immunotec) and mycobacterial culture [14]. These results indicate that dPCR is more sensitive than the other molecular and culture methods, and that dPCR could serve as a less invasive diagnostic tool for MTB infections [14]. In two other studies [15, 16] dPCR was used to detect major BSI Gram-negative pathogens in cfDNA isolated from plasma of critically ill patients. Shin et al. [15] developed a dPCR assay able to detect four major Gram-Negative pathogens and four common antimicrobial resistance (AMR) genes. Zheng et al. [16] work focused on only two of the most common MDR Gram-Negative pathogens. These assays report a time from sample collection to result of three to four hours and a detection limit of one Colony-forming Unit/ml of bacteria in the blood [15, 16]. The study by Shin et al. indicated that dPCR was also more sensible than qPCR [15]. Hu et al. [10] compared the detection of pathogens and AMR genes by dPCR with metagenomic Next-Generation Sequencing (mNGS) and with blood culture using samples from a cohort of septic patients with suspicion of BSIs. dPCR showed a great potential to identify the pathogens most commonly associated with BSIs as well as AMR genes, as it was faster and more sensible than mNGS and blood culture [10]. In these previous studies [10, 15, 16], clinical validation revealed that dPCR method was superior to blood culture in terms of specificity, sensitivity, and turnaround time, representing a promising method for the early and accurate diagnosis of BSIs [10, 15, 16]. Our group has established a ddPCR assay capable of detecting and quantifying the housekeeping genes of important nosocomial bacterial species in ICUs, such as *Klebsiella pneumoniae*, *Escherichia coli* and *Staphylococcus aureus* [17]. These assays are compatible with duplexing, show low replication variability and very low limit of detection (less than 1 pg of DNA) (Fig. 3). Ongoing work is focused on applying the developed ddPCR assays directly to blood and designing a panel that allows testing as many samples at the same time as possible.

The main limitation of dPCR compared with blood culture or mNGS, but not with qPCR, is that it can only detect the pathogens included in the dPCR panels. Nevertheless, the results obtained support that early identification of MDR pathogens by dPCR can improve treatment outcomes [10, 15, 16].

#### Fungal identification in blood

dPCR has been also tested for the detection of candidemia [18], showing again higher specificity and sensitivity than blood culture and qPCR (both methods yielding negative results in these patients). Detection of *Candida* spp in blood by dPCR allowed the implementation of the appropriated antifungal treatment, which translated into patients’ improvement, supporting the use of dPCR for the early diagnosis of candidemia [18].

#### Bacterial and fungal identification in other clinical samples

dPCR has also been applied to the detection of pathogens in clinical samples other than blood. For instance, Zhou et al. [19] tested dPCR to detect bacteria and fungi in pleural and peritoneal fluids of critically ill patients, comparing the obtained results with culture methods. Compared to the gold standard cultures of pleural and peritoneal fluids, dPCR showed a sensitivity of 96% and 93%, specificity of 87% and 60%, a positive predictive value of 92% and 87% and a negative predictive value of 93% and 75%, respectively. These results support dPCR as a rapid and sensitive alternative for the detection of pleural and peritoneal fungal infections [19].

#### Viral identification

dPCR has been also tested for viral diagnosis. Simms et al. [20] used commercial Severe acute respiratory syndrome coronavirus 2 (SARS-CoV-2) dPCR kits to detect this virus in a patient diagnosed with sepsis ten days after a renal transplant. SARS-CoV-2 was detected on the renal allograft and native lung tissue post-mortem by dPCR but not by qPCR [20] indicating a higher sensitivity of the former method. Two other studies confirmed the higher sensitivity of dPCR compared with qPCR to detect SARS-CoV-2 [21, 22] as they identified this virus in symptomatic cases and asymptomatic carriers with low viral copy number (less than 2,000 copies/mL) [22], being these cases negative by qPCR. In the study by Alteri et al., SARS-CoV-2 infection diagnosed by dPCR was confirmed by detection of SARS-CoV-2 IgG at a later stage of the illness [21].

#### Quantification of microbial burden to assess severity, prognosis and treatment guidance

The possibility to quantify microbial genes by dPCR makes it an ideal method to stratify severity, assess prognosis and guide treatment. Time to positivity of blood culture has been suggested as a surrogate marker of the bacterial load present in the blood and of poor clinical outcome in BSI [23–25]. Some studies have also associated persistent BSI, detected in follow-up blood cultures, as a marker for endovascular complications and mortality risk [26–28].

Two studies published by Ziegler et al. [29, 30] showed that dPCR could be used to quantify bacterial DNA, either using species-specific genes [29, 30] or 16S ribosomal DNA (rDNA) [30] in blood of critically ill patients. Results showed that a high initial 16S rDNA load was associated with both sepsis at admission and mortality, which indicates a potential clinical value for the quantification of bacterial DNA in blood at patient admission [30]. The monitoring of patients’ bacterial load during the course of BSI showed that non-survivors had significantly higher DNA loads than survivors. The authors also found that high DNA load tended to be associated to prolonged C-reactive protein elevation and lymphopenia. The benefit of monitoring clinical progression determining DNA load was further demonstrated by Bialasiewicz et al. [31]. In this study, the authors assessed the effectiveness of antimicrobial therapy by quantifying bacterial DNA load at day zero and five, showing a great decrease in DNA load over time [31].

Another study by Dickson et al. [32] used dPCR to quantify the bacterial DNA burden in the lung at ICU admission, by assessing the concentration of 16S rDNA in mini-bronchoalveolar lavage. This study showed that patients with the highest lung bacterial DNA burden at the baseline were less likely to be extubated and alive at 7, 14, 21, and 28 days than patients with low bacterial DNA burden [32].

dPCR has also demonstrated its value to assess severity and prognosis in severe viral infections. Goh et al. [33] quantified, by dPCR, the viral load of Epstein-Barr Virus (EBV) in the plasma of patients with sCAP-derived sepsis. The hypothesis was that EBV reactivation and viral load levels might be a useful biomarker of patient immunosuppression and prognosis. The authors found that EBV reactivation was common in septic patients, that EBV viral load increased over time, was associated to longer ICU stays and increased organ failure. These findings are consistent with the concept that viral reactivation is a consequence of immune compromise in sepsis [33]. dPCR has also demonstrated its potential for the detection and quantification of SARS-CoV-2 in plasma of COVID-19 patients [34–40]. The percentage of patients with SARS-CoV-2 RNAaemia varies amongst studies (74% to 92%) [34, 35, 38], but available evidence supports that SARS-CoV-2 RNAaemia correlates with disease severity (RNAaemia prevalence ranges from 2% in outpatients, 27–53% in mild-to-moderate patients and 78–88% in critically ill patients) [34, 36, 40]. Viral RNA load in plasma correlates with key signatures of dysregulated host responses, suggesting a major role of uncontrolled viral replication in the pathogenesis of critical illness caused by SARS-CoV-2 [35, 36, 39]. It is also a predictor of severe outcome [35, 37, 39]. dPCR has been compared with qPCR for the detection of SARS-CoV-2 RNAaemia, with one study showing that dPCR is more sensitive than qPCR [37] and the other showing that both could indifferently be used [38]. This disagreement is probably related with the qPCR method used for the detection of SARS-CoV-2 RNAemia.

#### Microbial ecology studies

Chanderraj et al. [41] used dPCR to quantify bacterial DNA in rectal swabs from hospitalized patients and compared bacterial density and bacterial community composition (using 16S rDNA gene and amplicon sequencing), with clinical exposures (e.g., antibiotics and comorbidities), and subsequent risk of culture-confirmed extra-intestinal infection. This study showed that bacterial DNA density was associated to clinical comorbidities and age, and exposure to piperacillin-tazobactam. It was also predictive of infection during hospitalization [41]. In the same line, Brooks et al. [42] quantified bacterial DNA load by dPCR (through 16S rDNA), to evaluate if the bacterial density in an ICU environment influenced the establishment of the microbiome in hospitalized premature infants. The authors showed that bacterial DNA load and diversity varied between surfaces. Room-specific microbiome signatures were detected, suggesting that the microbes seeding ICU surfaces are sourced from reservoirs within the room, possible shaping hospitalized infants gut microbiome [42].

### Applications of dPCR targeting host response

Results coming from high-throughput technologies such as microarrays or next-generation sequencing, which are able to analyse the entire human transcriptome, have revealed the existence of specific host response signatures potentially useful to improve diagnosis [43], severity stratification and prognosis assessment [44, 45] of severe infection. dPCR is making real the promise of translating these signatures into the clinical practice. A work from our group was pioneer in exploring the potential use of dPCR to diagnose sepsis, evidencing that the gene expression ratio between the constant region of the mu heavy chain of IgM and CD20 yielded an area under the receiver operating curve (AUROC) of 0.72 to differentiate sepsis from systemic inflammatory response syndrome (SIRS) [46]. In turn, Almansa et al. evidenced that the transcriptomic ratios between matrix metalloproteinase-8 (MMP8) or Lipocalin 2 (LCN2), which are two genes coding for the proteins contained in the neutrophil granules, with the major histocompatibility complex class II, DR alpha molecule (HLA-DRA), yielded AUROCs > 0.89 to differentiate between sepsis and SIRS [47]. The combination of gene expression profiling by dPCR with standard biomarkers commonly used in the clinical practice is also an exciting avenue of research, already explored in another work from Almansa et al., which evidenced that the combination of procalcitonin and HLA-DRA expression levels outperformed the former biomarker to detect sepsis. In this work, procalcitonin yielded an AUROC of 0.80 and the ratio Procalcitonin/HLA-DRA of 0.85 [48]. In a small study, Link et al. explored the potential of microRNA (miRNA) expression quantification by dPCR to diagnose sepsis. These authors found that miR-26b-5p yielded an AUROC of 0.80 to differentiate critically ill patients with sepsis from those with no sepsis [49]. dPCR has been also employed to evaluate the magnitude of the biological processes occurring during sepsis that are difficult to quantify with the currently available methods. For example, in a cohort of patients with infection, sepsis or septic shock, Martin-Fernandez et al. evidenced a progressive increase in the expression levels of emergency granulopoiesis related genes with severity [50]. Another signature of sepsis and sCAP is the depressed expression of those genes involved in the immunological synapse between antigen-presenting cells and T cells [51]. Menéndez et al. demonstrated that dPCR is an useful method to evidence the depressed expression of three of these genes [HLA-DRA, CD40 Ligand (CD40LG) and CD28] in patients with CAP presenting with organ failure [52], while Almansa et al. evidenced that profiling the expression levels of immunological synapse genes was also useful to identify VAP, yielding AUROCs of 0.82 for CD40LG, 0.79 for inducible T cell costimulator (ICOS), 0.78 for CD28 and 0.74 for CD3E [53]. Regarding prognosis, Busani et al. used dPCR to evidence the potential role of mitochondrial DNA as predictor of mortality in patients with septic shock due to MDR bacteria [54]. In turn, Almansa et al. showed that gene expression levels of HLA-DRA quantified by dPCR were an independent predictor of mortality in sepsis [47]. Cajander et al. had already proposed to use expression levels of this gene to identify those sepsis patients that could benefit from immunostimulatory drugs [55]. More recently, using dPCR, Bruneau et al.revealed that expression levels of a circulating ubiquitous RNA (RNase P) correlated with disease severity, invasive mechanical ventilation status and survival in patients with COVID-19 [40]. Also in severe COVID-19, Sabbatinelli et al. found that low levels in plasma of the inflamm-aging associated miRNA miR-146a were associated with no response to tocilizumab [56]. Metabolomics is a relatively new “-omic” that has demonstrated its use in the management of septic patients (e.g. lactate). The combination of this “-omics” approach with the transcriptomics markers measured by dPCR might be useful to determine patient severity, predict the need for mechanical ventilation and mortality [57].

## Conclusions

This review evidences the potential of dPCR as a useful tool that could contribute to improve the diagnosis and clinical management of infection in critical care medicine. Although most of the published works consist of pilot/ exploratory studies, they show the potential of dPCR, supporting the development of further, larger studies aimed to validate the use of this technology in this field.

## Supplementary Information

**Additional file 1: Table S1.** Comparison of techniques to detect microorganisms that can be employed to diagnose the most common infections affecting critically ill patients. Table containing description of emerging and current techniques to detect microorganism that can be applied to the most common critically ill patients’ infections, including the most important advantages and disadvantages.

## Data Availability

The dataset supporting the conclusions of this article is included within the article (and its additional file).

## Abbreviations

AMR: Antimicrobial resistance genes
AUROC: Area under the receiver operating curve
BSIs: Bloodstream infections
CD40LG: CD40 Ligand
cfDNA: Cell-free deoxyribonucleic acid
COVID-19: Coronavirus disease 2019
ddPCR: Droplet digital polymerase chain reaction
DNA: Deoxyribonucleic acid
dPCR: Digital polymerase chain reaction
EBV: Epstein-Barr Virus
HLA-DRA: Major histocompatibility complex class II, DR alpha molecule
ICOS: Inducible T Cell Costimulator
ICU: Intensive care unit
IgG: Immunoglobulin G
IgM: Immunoglobulin M
LCN2: Lipocalin 2
MDR: Multidrug-resistance
miRNA: Micro ribonucleic acid
MMP8: Matrix metalloproteinase-8
mNGS: Metagenomic Next-Generation Sequencing ()
MTB: Mycobacterium tuberculosis
mtDNA: Mitochondrial DNA
PCR: Polymerase chain reaction
PCT: Procalcitonin
qPCR: Real-time polymerase chain reaction
rDNA: Ribosomal deoxyribonucleic acid
RNA: Ribonucleic acid
SARS-CoV-2: Severe acute respiratory syndrome coronavirus 2
sCAP: Severe community acquired pneumonia
SIRS: Systemic inflammatory response syndrome
VAP: Ventilator-associated pneumonia
