# Supplementary material for: Digital PCR applications for the diagnosis and management of infection in critical care medicine
Source: Crit Care. 2022 Mar 21;26:63. doi: 10.1186/s13054-022-03948-8 (PMC8935253; doi:10.1186/s13054-022-03948-8)
Supplement: Supplementary file 1 — Additional file 1: Table S1. Comparison of techniques to detect microorganisms that can be employed to diagnose the most common infections affecting critically ill patients. Table containing description of emerging and current techniques to detect microorganism that can be applied to the most common critically ill patients’ infections, including the most important advantages and disadvantages. [file 13054_2022_3948_MOESM1_ESM.docx]

**Table S1. Comparison of diagnostic techniques used to detect microorganism that can be applied to the most common critically ill patients’ infections.**

| **Method** | **Sample** | **Technology** | **Time from sample to result** | **Advantages** | **Disadvantages** | Reference |
| --- | --- | --- | --- | --- | --- | --- |
| **dPCR** | Blood, plasma and other clinical samples | dPCR | 3-4h | - Significantly reduces time from sample to results;  - ±30min hands-on time;  - High sensitivity and specificity;  - Flexible/open system;  - Detection of microorganisms and AMR genes;  - No standard curve required;  - Not affected by PCR inhibitors. | - Specialized training;  - Unable to distinguish between viable and non-viable microorganisms;  - High cost (particularly the initial investment). |  |
| **Quick-FISH** | Positive blood culture | FISH | 30min* | - Significantly reduces time from sample to results;  - ±5 min hands-on time;  - No specialized training;  - High sensitivity and specificity (>95%);  - Flexible/open system;  - Low cost. | - Requires positive cultures (blood);  - Closed system;  - Detects a limited number of microorganisms;  - Contingent upon correct interpretation of a Gram stain;  - Requires a microorganism concentration of at least 10^5^ CFU/mL for detection;  - No AMR gene detection. | 22493336 |
| **PNA-FISH** | Positive blood and peritoneal fluid cultures | FISH | 2h* | - Significantly reduces time from sample to results;  - ±5 min hands-on time;  - No specialized training;  - High sensitivity and specificity (>95%);  - Flexible/open system;  - Low cost. | - Requires positive cultures (blood);  - Closed system;  - Detects a limited number of microorganisms;  - Contingent upon correct interpretation of a Gram stain;  - Requires a microorganism concentration of at least 10^5^ CFU/mL for detection;  - No AMR gene detection. | 14510184 |
| **Eazyplex PneumoBug** | BAL, bronchial and tracheal samples | LAMP | 25min* | - Significantly reduces time from sample to results;  - ±5 min hands-on time;  - No specialized training;  - Bench size system;  - No DNA extraction. | - Unable to distinguish between viable and non-viable microorganisms;  - High cost;  - Closed system;  - Detects a limited number of microorganisms;  - No AMR gene detection. |  |
| **Eazyplex BloodScreen** | Positive blood culture | LAMP | 20min* | - Significantly reduces time from sample to results;  - ±5 min hands-on time;  - No specialized training;  - High specificity (>90%) depending on the target;  - Bench size system;  - No DNA extraction. | - Requires positive cultures (blood);  - High cost;  - Closed system;  - Detects a limited number of microorganisms;  - No AMR gene detection;  - Low sensitivity (>67%) depending on the target | 34807364 |
| **ID-Now Abbot** | NP swab | LAMP | <1h | - Significantly reduces time from sample to results;  - ±5 min hands-on time;  - High specificity (>95%);  - Bench size system. | - Unable to distinguish between viable and non-viable microorganisms;  - Low sensitivity (79%);  - Only detects virus. | 33913533 |
| **eazyplex SARS-CoV-2** | NP swab | LAMP | 25min | - Significantly reduces time from sample to results;  - ±10 min hands-on time;  - No specialized training;  - Bench size system;  - No RNA extraction. | - Unable to distinguish between viable and non-viable virus;  - High cost;  - Closed system;  - Only accurate in high viral load samples (>74% sensitivity for samples with Ct<35). | 33836452 |
| **MALDI-TOF** | Positive blood, urine, CSF cultures | Mass spectrometry | <1h* | - Significantly reduces time from sample to results;  - ±10 min hands-on time;  - No specialized training;  - High specificity (>95%);  - Flexible/open system;  - Low cost. | - Requires positive cultures (blood);  - High initial investment;  - Closed system;  - Detects a limited number of microorganisms;  - Requires a microorganism concentration of at least 10^5^ CFU/mL for detection;  - Low sensitivity;  - Relies on databases;  - Does not detect polymicrobial infections. | 34361974 |
| **Verigene (BC-GP,**  **BC-GN)** | Positive blood culture | Microarray | <2h* | - Significantly reduces time from sample to results;  - ±5 min hands-on time;  - No specialized training;  - Detection of microorganisms and AMR genes. | - Requires positive cultures (blood);  - Closed system;  - Detects a limited number of microorganisms;  - Low through-put;  - Low sensitivity;  - Does not detect polymicrobial infections. | 30477953 |
| **Verigene RP** | NP swab | Microarray | <2h* | - Significantly reduces time from sample to results;  - ±5 min hands-on time;  - No specialized training. | - Closed system;  - Detects a limited number of microorganisms;  - Low through-put;  - Does not detect polymicrobial infections. |  |
| **Accelerate Pheno System** | Positive blood culture | FISH; Morphokinetic cellular analysis | - 90min^*^ (identification)  -7h* (AMR detection) | - Significantly reduces time from sample to results;  - ±5 min hands-on time;  - No specialized training;  - High specificity (>87%);  - Detection of microorganisms and AMR genes;  - Phenotypic antibiotic susceptibility detection;  - Detects polymicrobial infections. | - Requires positive cultures (blood);  - High cost;  - Closed system;  - Detects a limited number of microorganisms and AMR genes. |  |
| **FilmaArray Respiratory panel** | BAL | Nested qPCR | 1h | - Significantly reduces time from sample to results;  - ±5 min hands-on time;  - No specialized training;  - On-demand access. | - Unable to distinguish between viable and non-viable virus;  - High cost;  - Closed system;  - Detects a limited number of virus;  - Low through-put. | 29018819 |
| **FilmArray Bloodstream panel** | Positive blood culture | nested qPCR | 1h* | - Significantly reduces time from sample to results;  - ±5min hands-on time;  - No specific training;  - High sensitivity (>99%);  - Detection of microorganisms and AMR genes;  - On-demand access. | - Requires positive cultures (blood);  - High cost;  - Closed system;  - Detects a limited number of microorganisms and AMR genes;  - Low through-put. | 32305272 |
| **FilmArray Pneumonia panel** | Esputum, BAL, miniBAL, endotracheal aspirate | nested qPCR | 1h | - Significantly reduces time from sample to results;  - ±5min hands-on time;  - No specific training;  - Detection of microorganisms and AMR genes;  - On-demand access;  - Semi-quantitative results for some targets. | - Unable to distinguish between viable and non-viable microorganisms;  - High cost;  - Closed system;  - Detects a limited number of microorganisms and AMR genes;  - Low sensitivity for some targets;  - Low through-put;  - Difficult to interpret results (no quantification - important to distinguish between infection and colonization). | 33130507 |
| **Metagenomics** | Blood sample | NGS | 1d | - Significantly reduces time from sample to results;  - Flexible/open system;  - Detection of microorganisms and AMR genes;  - No limitation on the number of microorganism detected, including no-cultivable pathogens. | - Specialized training;  - Unable to distinguish between viable and non-viable microorganisms;  - >5h hand-on time;  - High cost;  - Relies on databases;  - Difficult to interpret results. |  |
| **SeptiTest** | Blood sample | PCR  +  sequencing | 8-12h | - Significantly reduces time from sample to results;  - High sensitivity (>87%) and specificity (86%);  - Semiopen system (detects >300 microorganisms);  - Detects polymicrobial infections. | - Specialized training;  - Unable to distinguish between viable and non-viable microorganisms;  - High cost;  - Closed system;  - Difficult to interpret results;  - Low through-put. |  |
| **Abbott Plex-ID/IRIDICA** | Blood and other clinical samples | PCR  +  Mass Spectrometry | 7h | - Significantly reduces time from sample to results;  - High negative predictive value (97%);  - High multiplexing capability;  - Detects polymicrobial infections;  - Low detection limit (4-16 CFU/mL). | - Specialized training;  - Unable to distinguish between viable and non-viable microorganisms;  - High cost;  - Closed system;  - Detects a limited number of microorganisms;  - No AMR gene detection.  - Low sensitivity (81%) and specificity (69%);  - Relies on databases. | 28427796 |
| **LightCycler SeptiFast** | Blood sample | qPCR | 3-6h | - Significantly reduces time from sample to results;  - High specificity (92%);  - Covers 90% of most common BSI pathogens;  - Low detection limit (3-30 CFU/mL for bacteria and 100 CFU/mL for fungi). | - Specialized training;  - Unable to distinguish between viable and non-viable microorganisms;  - High cost;  - Closed system;  - Detects a limited number of microorganisms;  - Detects just 1 AMR gene;  - Low sensitivity (75%);  - Low through-put. | 28427796 |
| **Magicplex Sepsis Real-Time** | Blood sample | qPCR | 3-6h | - Significantly reduces time from sample to results;  - High specificity (92%). | - Specialized training;  - Unable to distinguish between viable and non-viable microorganisms;  - High cost;  - Closed system;  - Detects a limited number of microorganisms and AMR genes;  - Low sensitivity (±70%). | 28427796 |
| **GeneXpert** | NP swab, plasma | qPCR | 30min-1h | - Significantly reduces time from sample to results;  - ±5min hands-on time;  - No specific training;  - On-demand access. | - Unable to distinguish between viable and non-viable microorganisms;  - High cost;  - Closed system;  - Detects a limited number of microorganisms and AMR genes;  - Low through-put. |  |
| **T2Dx** | Blood sample | T2MR  (miniaturized magnetic resonance) | 5h | - Significantly reduces time from sample to results;  - ±5min hands-on time;  - High sensitivity and specificity (90%);  - High negative predictive value (±100%);  - On-demand access. | - Unable to distinguish between viable and non-viable microorganisms;  - High cost;  - Closed system;  - Detects a limited number of microorganisms and AMR genes;  - Low through-put. | 31083728 |

* After positive blood culture.

**Abbreviations:** AMR – Antimicrobial resistance, FISH - Fluorescence in situ hybridization, PNA - Peptide nucleic acid probes, BAL – Bronchoalveolar Lavage, LAMP - Loop-mediated isothermal amplification, MALDI-TOF - Matrix-assisted laser desorption ionization time-of-flight, NP – Nasopharyngeal, BC – Blood culture, GP – Gram-Positive, GN – Gram-Negative, RP - Respiratory Pathogen, qPCR – real time polymerase chain reaction, NGS – Next-generation sequencing, PCR - polymerase chain reaction.
